# Charakteristika älterer im Vergleich zu jüngeren Notfallpatienten: Analyse von über 356.000 erfassten Besuchen des AKTIN-Notaufnahmeregisters

**DOI:** 10.1007/s00063-022-00968-8

**Published:** 2022-11-04

**Authors:** Katharina Langhoop, Kirsten Habbinga, Felix Greiner, Falk Hoffmann, AKTIN-Notaufnahmeregister, Markus Wehler, Sabine Blaschke, Tobias Hofmann, Benjamin Lucas, Caroline Grupp, Christian Pietsch, Oliver Horn, Heike Höger-Schmidt, Harald Dormann, Greta Ullrich, Kirsten Habbinga, Thomas Henke, Tobias Schilling, Bernadett Erdmann, Eckart Wetzel, Markus Baacke, Rupert Grashey, Rainer Röhrig, Raphael Majeed, Jonas Bienzeisler, Felix Walcher, Wiebke Schirrmeister, Ronny Otto

**Affiliations:** 1 Department für Versorgungsforschung, Fakultät VI – Medizin und Gesundheitswissenschaften, Carl von Ossietzky Universität Oldenburg, Ammerländer Heerstraße 114–118, 26129 Oldenburg, Deutschland; 2 Medizinischer Campus Universität Oldenburg, Pius-Hospital Oldenburg, Oldenburg, Deutschland; 3 Universitätsklinik für Unfallchirurgie, Otto-von-Guericke-Universität Magdeburg, Magdeburg, Deutschland; 4 Zentralinstitut für Arbeitsmedizin und Maritime Medizin (ZfAM), Universitätsklinikum Hamburg-Eppendorf (UKE), Hamburg, Deutschland

**Keywords:** Notaufnahme, Stationäre Versorgung, Versorgungsforschung, Geriatrie, Demografischer Wandel, Emergency department, In-house treatment, Health services research, Geriatrics, Demographic change

## Abstract

**Hintergrund:**

Ziel ist es, bundesweit und klinikübergreifend altersspezifische Unterschiede in den Charakteristika insbesondere von älteren Notfallpatienten herauszuarbeiten.

**Methodik:**

Aus 11 sowohl universitären als auch außeruniversitären Notaufnahmen, angeschlossen an das AKTIN (Aktionsbündnis für Informations- und Kommunikationstechnologie in Intensiv- und Notfallmedizin) -Notaufnahmeregister, wurden für das Kalenderjahr 2019 Daten aller Notfallpatienten im Mindestalter von 18 Jahren analysiert. Neben demografischen Daten wurden Variablen wie Zu‑/Einweiser, Transportart, Stufe der Ersteinschätzung, Diagnosen, Aufenthaltsdauer und Verlegungsart erfasst und nach Altersgruppen sowie dezidiert nach jüngeren (18–64 Jahre) und älteren Patienten (65+ Jahre) verglichen.

**Ergebnisse:**

Eingeschlossen wurden Daten von 356.354 Patienten (39,1 % davon  65+ Jahre). Im Vergleich zu jüngeren werden ältere Notfallpatienten eher rettungsdienstbegleitet (15,4 % vs. 34,3 %) und fast doppelt so häufig notarztbegleitet (6,4 % vs. 12,2 %). Mit zunehmendem Alter nimmt die Therapiedringlichkeit zu, 47,1 % der Jüngeren und 66,1 % der Älteren wurden als gelb, orange oder rot eingestuft. Gleichzeitig sind bei 65+-Jährigen internistische Erkrankungen (22,5 % vs. 38,8 %) als auch stationäre Aufnahmen (27,5 % vs. 60,3 %) sowie direkte Verlegungen auf Intensivstation (4,5 % vs. 11,9 %) deutlich häufiger als bei den jüngeren Notfallpatienten.

**Schlussfolgerung:**

Etwa 40 % aller erwachsenen Notfallpatienten sind 65+ Jahre alt. Sie sind im Vergleich zu Jüngeren dringlicherer behandlungsbedürftig und werden deutlich häufiger stationär aufgenommen. Bei den älteren Patienten sind häufiger internistische Krankheitsbilder die führenden Notaufnahmediagnosen.

**Zusatzmaterial online:**

Zusätzliche Informationen sind in der Onlineversion dieses Artikels (10.1007/s00063-022-00968-8) enthalten.

## Abstract (en)

**Background:**

This nationwide study aims to analyze age-specific differences and characteristics of emergency patients with a special focus on older patients.

**Method:**

In 2019, data were obtained from 11 emergency departments (EDs), all part of the German Emergency Department Data (AKTIN) registry. All patients 18 years and older visiting the EDs were included. In addition to demographic data, variables such as referral, type of transport, primary assessment, diagnoses, length of stay and type of transfer were recorded and compared by age group and specifically by younger (18–64 years) and older patients (65+ years).

**Results:**

Data from 356,354 patients (39.1% were aged 65+ years) were included. Compared to younger patients, older ED patients were more likely to be accompanied by emergency medical services (15.4 vs. 34.3%) and almost twice as often by an emergency physician (6.4 vs. 12.2%). The need for treatment increased with age; 47.1% of younger and 66.1% of older people were classified as yellow, orange or red. The proportion of patients with internal diseases was higher for patients 65+ years (22.5 vs. 38.8%). Older patients were more often hospitalized (27.5 vs. 60.3%) and were more frequently transferred to an intensive care unit (4.5 vs. 11.9%).

**Conclusion:**

About 40% of adult emergency patients are 65+ years. They require more urgent treatment and are more often hospitalized than younger patients. In older patients, internal diseases were more common.

## Einleitung

Die Notfallversorgung stellt für Patienten mit akutem Behandlungsbedarf einen essenziellen Sektor im Gesundheitssystem dar [1, 2]. Dieser wird in Deutschland über den vertragsärztlichen Bereitschaftsdienst, die Notaufnahmen der Krankenhäuser sowie den Rettungsdienst organisiert, für die jeweils unterschiedliche gesetzgeberische Zuständigkeiten und rechtliche Vorgaben gelten [3].

Deutsche Notaufnahmen stehen in den letzten Jahren zunehmend im Fokus politischer Reformbemühungen. Als Gründe dafür werden unter anderem Überfüllung und Überlastung u. a. bedingt durch eine ineffiziente Patientensteuerung, verändertes Patientenverhalten bzw. die Erwartung einer besseren und schnelleren Versorgung sowie regional unterschiedliche Strukturen genannt [3–5]. Eine weitere nicht unerhebliche Herausforderung für die pflegerische und medizinische Versorgung in Notaufnahmen stellt der demografische Wandel dar [6–8]. So ist es aufgrund der häufig mit dem Alter einhergehenden Phänomene, wie Multimorbidität sowie Polypharmazie, funktionellen und kognitiven Einschränkungen, oft schwierig, akute von bereits bestehenden Problemen abzugrenzen [9]. Hinzu kommen atypische Krankheitspräsentationen sowie oft unklare Zuständigkeiten und in der Regel zu geringe geriatrische Expertise in Notaufnahmen [9–11]. Hingegen kennzeichnen jüngere Notfallpatienten häufig banale Vorstellungsgründe [3, 12]. Sie suchen Notaufnahmen teils aufgrund der Verfügbarkeit und des niederschwelligen Zugangs auf [13, 14].

Um zukünftige Strukturen der Notfallversorgung gestalten zu können, sind belastbare Daten zur Anzahl und Charakteristika von Notfallpatienten sowie zu altersspezifischen Unterschieden essenziell [15, 16]. Schätzungen zufolge werden jährlich zwischen 21 und 30 Mio. Patienten in deutschen Notaufnahmen behandelt [17, 18]. Aufgrund der demografischen Entwicklung wird eine Zunahme und weitere Verschiebung des Altersspektrums erwartet [19, 20].

Einzelne Studien, wie die von Trentzsch et al. analysierten zwar dezidiert unterschiedliche Charakteristika zu Patienten aus 14 Notaufnahmen, jedoch ohne altersspezifische Betrachtung [21], während Rygiel et al. deutliche Unterschiede zwischen jüngeren und älteren Notfallpatienten nachwiesen [6], diese Studie jedoch ausschließlich eine universitäre Notaufnahme eingeschlossen hatte. Auch Rauch et al. präsentierten Ergebnisse aus einer einzelnen Notaufnahme, analysierten jedoch ausschließlich Unterschiede zwischen gebrechlichen und nichtgebrechlichen Patienten [16]. Insgesamt existieren deutschlandweit kaum umfassende Daten, wie sich ältere im Vergleich zu jüngeren Notfallpatienten in Bezug auf Einweisungsart, Diagnosen und die weitere Versorgung unterscheiden.

Ziel dieser explorativen Analyse ist es daher, deutschlandweit zu untersuchen, wie sich die Charakteristika von erwachsenen Notaufnahmebesuchen altersgruppenspezifisch unterscheiden.

## Material und Methodik

### Datenbasis

Verwendet wurden Routinedaten des AKTIN (Aktionsbündnis für Informations- und Kommunikationstechnologie in Intensiv- und Notfallmedizin) -Notaufnahmeregisters, das basierend auf dem standardisierten DIVI (Deutsche Interdisziplinäre Vereinigung für Intensiv- und Notfallmedizin) -Datensatz Notaufnahme strukturierte Daten zu allen Notfallpatienten der beteiligten Kliniken erhebt. Die Daten werden elektronisch im Notaufnahmeinformationssystem erfasst und in einem krankenhauseigenen Data Warehouse gespeichert. Nach lokaler Erteilung der Freigabe zur Datennutzung erfolgt die anonymisierte Versendung über den AKTIN-Broker zur Auswertung [22].

Zum Zeitpunkt der Studie beteiligten sich deutschlandweit 17 Kliniken am Notaufnahmeregister, 14 davon gaben ihre Zustimmung zur Datenfreigabe. Aufgrund unvollständiger Datensätze wurden 3 Kliniken ausgeschlossen. Die verbliebenen 11 Kliniken aus 5 Bundesländern verteilen sich auf eine Klinik mit Basisnotfallversorgung sowie 5 mit erweiterter und weitere 5 mit umfassender Notfallversorgung (Versorgungsstufen gemäß GBA-Beschluss; [23]). Die Bettenanzahl der teilnehmenden Kliniken beträgt im Median 547 (von 184 bis 1765 je Klinik). Details zeigt eTabelle 1.

### Studienpopulation

Eingeschlossen wurden alle Patienten im Mindestalter von 18 Jahren, die im Zeitraum vom 01.01.2019 bis zum 31.12.2019 die Notaufnahme einer der beteiligten Kliniken aufsuchten.

### Variablen

Folgende Variablen wurden ausgewertet: a) demografische Daten, b) Zu‑/Einweiser, c) Transportart, d) Ersteinschätzung gemäß Manchester Triage System (MTS; [24]) oder Emergency Severity Index (ESI) sowie e) alle Notaufnahmediagnosen nach ICD (international classification of diseases)-10, die wir gruppiert nach Ramroth ausgewertet haben [25]*. *Darüber hinaus standen uns Informationen über Zeitparameter wie f) Aufnahmedatum und -zeitpunkt, g) Dauer bis zum ersten Arztkontakt sowie h) Aufenthaltsdauer in der Notaufnahme und i) Verlegungs- bzw. Entlassungsart zur Verfügung. Weitere Details zeigen eTabelle 2 und 3.

### Statistische Analysen

Es wurden deskriptive Auswertungen stratifiziert nach Altersgruppen (18–34, 35–44, 45–54, 55–64, 65–74, 75–84, 85–94 und 95+ Jahre) sowie zusammengefasst in Jüngere (18–64 Jahre) und Ältere (65+ Jahre) und nach Geschlecht (Männer, Frauen) durchgeführt. Bei den Diagnosen wurden alle als „gesichert“ kodierte Diagnosen, teils auch mehrere pro Fall, berücksichtigt.

Das AKTIN-Notaufnahmeregister wurde durch die Ethikkommission der Otto-von-Guericke-Universität Magdeburg an der medizinischen Fakultät positiv beurteilt (Votum160/15) und ist im Deutschen Register Klinischer Studien registriert (DRKS00009805). Unser Antrag auf Datenauswertung wurde durch das wissenschaftliche Gremium von AKTIN genehmigt (Notaufnahmeregister-Projekt-ID: 2019-005). Die Datenaufbereitung und -auswertung erfolgte durch das AKTIN Trusted Data Analyzing Center mit Übermittlung aggregierter Ergebnismengen.

## Ergebnisse

### Baselinecharakteristika

Im Studienzeitraum schlossen wir insgesamt 356.354 Notfallpatienten ein, davon waren 216.894 (60,9 %) 18–64 Jahre (jüngere) und 139.460 (39,1 %) 65+ Jahre (ältere).

### Zu‑/Einweiser, Transportart, Triage

**Figure 1:**
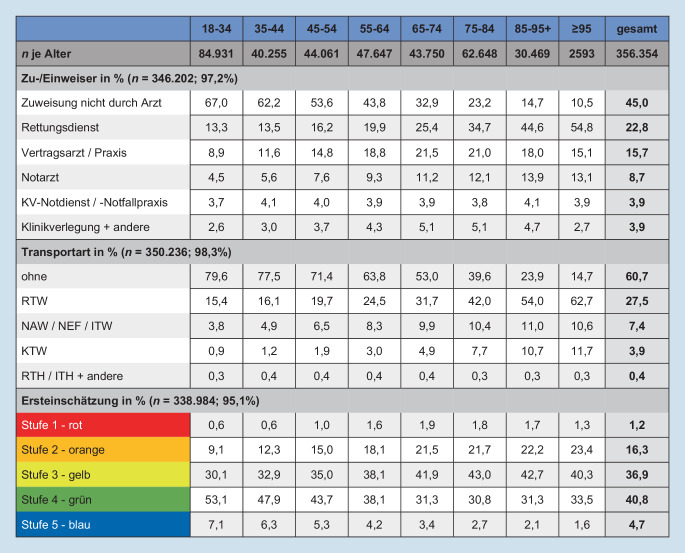

Insgesamt kamen 45 % aller Patienten als Selbsteinweiser und 60,7 % ohne qualifiziertes Transportmittel in die Notaufnahme. Mit zunehmendem Alter sanken diese Anteile deutlich (Abb. 1). Im Vergleich zu jüngeren wurden ältere Notfallpatienten eher rettungsdienst- (15,4 % vs. 34,3 %) bzw. notarztbegleitet (6,4 % vs. 12,2 %) vorgestellt (eTabelle 2).

Insgesamt wurden 45,5 % aller Notfallpatienten als nicht dringlich (grün, blau) ersteingeschätzt, wobei dieser Anteil mit steigendem Alter deutlich abnahm (von 60,2 % bei 18–34 auf 35,1 % bei 95+ Jahren).

### Notaufnahmediagnose

Zu 300.127 (84,2 %) Notaufnahmebesuchen lag mindestens eine Diagnose vor. Insgesamt 29,5 % aller Diagnosen kamen aus dem internistischen Spektrum, mit zunehmendem Alter deutlich häufiger. Verletzungen und Intoxikationen kamen bei 26,8 % aller Diagnosen vor, 2‑gipflig präsentierend in den Altersgruppen 18–34 und 95+ Jahre (jeweils ca. 35 %). Bei den 75- bis 84- und 85- bis 94-Jährigen kamen internistische Notaufnahmediagnosen mit jeweils nahezu 40 % deutlich häufiger vor als Verletzungen und Intoxikationen. Im Vergleich zu jüngeren wurden ältere Notfallpatienten eher internistisch vorgestellt (22,5 % vs. 38,8 %). Ein kardiovaskuläres Krankheitsbild in der Notaufnahme war bei den Älteren 2,5-mal sowie endokrine, Ernährungs- und Stoffwechselerkrankungen 3‑mal häufiger als bei den Jüngeren. Zerebrovaskuläre Krankheitsbilder waren im Vergleich fast 3,5-mal häufiger bei den Älteren (Tab. 1, eTabelle 3).

**Table 1:** 

ICD-Gruppen	18–34	35–44	45–54	55–64	65–74	75–84	85–94	95+	Gesamt
*n* der Notaufnahmediagnosen^a^	73.082	35.166	39.701	45.075	43.874	64.831	32.063	2663	336.455
*Internistische Erkrankungen*	*17,3*	*19,6*	*24,9*	*31,0*	*38,1*	*39,1*	*39,4*	*34,6*	*29,4*
Kardiovaskuläre Erkrankungen *(I00–I52; I70–I99)*	2,1	4,1	7,5	11,0	14,2	15,4	14,5	10,7	9,6
Infektionen *(A00–B99)*	6,4	5,7	5,6	6,2	7,7	8,3	8,9	9,0	7,0
Gastrointestinale Erkrankungen *(K00–K93)*	5,4	6,3	6,8	7,0	6,9	6,3	6,3	6,2	6,3
Endokrine, Ernährungs- und Stoffwechselerkrankungen *(E00–E90)*	0,9	1,1	1,7	2,6	3,6	4,7	6,3	6,2	2,9
Respiratorische Erkrankungen *(J00–J99)*	2,3	1,9	2,0	2,4	3,3	2,7	2,5	2,0	2,5
Neubildungen *(C00–D48)*	0,2	0,5	1,1	1,8	2,4	1,6	0,9	0,4	1,2
*Andere Erkrankungen (D50–D89; L00–L99; N00–N99; O00–O99; P00–P96;R00–R99)*	*32,0*	*32,0*	*29,2*	*28,4*	*28,2*	*25,9*	*22,1*	*19,4*	*28,5*
Verletzungen und Intoxikation *(S00–T98)*	36,9	30,9	28,1	23,8	18,3	20,2	25,8	34,4	26,8
*Muskuloskelettale Erkrankungen (M00–M99)*	*6,0*	*8,2*	*7,9*	*6,8*	*5,4*	*4,8*	*3,7*	*3,3*	*6,0*
Zerebrovaskuläre Erkrankungen *(I60–I69)*	0,3	1,1	2,6	4,2	6,0	6,6	6,0	5,4	3,7
*Erkrankungen des Nervensystems und der Sinnesorgane (G00–G99; H00–H59; H60–H95)*	*3,6*	*3,9*	*3,5*	*3,1*	*2,7*	*2,4*	*1,7*	*1,2*	*3,0*
Psychische und Verhaltensstörungen *(F00–F99)*	3,9	4,4	3,9	2,6	1,3	1,1	1,2	1,6	2,6

*ICD* „international classification of diseases“, Gruppierung nach Ramroth

^a^mehrere Diagnosen pro Fall möglich

### Prozesszeiten und -parameter

Zwischen 08.00 und 20.00 Uhr kamen 73 % aller Notaufnahmepatienten in die Notaufnahme (Jüngere: 69,8 %; Ältere: 76,7 %).

Insgesamt fand bei 70,9 % aller Notfallpatienten innerhalb der ersten, bei 91,3 % innerhalb der ersten 2 h der erste Arztkontakt statt. Im Vergleich zu Jüngeren hatten Ältere einen früheren Arztkontakt. Knapp 30 % aller Patienten wiesen eine Aufenthaltsdauer von über 4 h in der Notaufnahme auf. Mit zunehmendem Alter stieg dieser Anteil deutlich an (18–34 Jährige: 19,5 % vs. 95+-Jährige: 37,0 %). Im Vergleich zu Jüngeren hielten sich Ältere länger in der Notaufnahme auf (Tab. 2, eTabelle 2). Während 7,1 % der Älteren bis zu 1 h in der Notaufnahme verbrachten, blieben 37,5 % über 4 h. (eTabelle 2, 6).

### Verlegungs‑/Entlassungsart

Insgesamt lag die stationäre Aufnahmequote bei mehr als 40 %, darunter 7,4 % Direktverlegungen auf die Intensivstation. Im Vergleich zu jüngeren blieben ältere Notfallpatienten häufiger stationär (27,5 % vs. 60,3 %) und wurden fast 3‑mal häufiger direkt auf Intensivstationen verlegt (4,5 % vs. 11,9 %; Tab. 2, eTabelle 2).

**Table 2:** 

Altersgruppe		18–34	35–44	45–54	55–64	65–74	75–84	85–94	95+	Gesamt
*Dauer bis zum ersten Arztkontakt in % (n* *=* *229.219*^a^*)*										
Bis 1 h		68,4	68,5	70,2	71,1	71,8	73,1	74,7	74,7	70,9
> 1 bis 2 h		22,1	22,3	20,9	20,0	19,6	18,7	17,8	18,7	20,4
> 2 bis 3 h		7,1	6,9	6,7	6,5	6,1	6,1	5,6	5,0	6,5
> 3 bis 4 h		2,4	2,3	2,2	2,3	2,4	2,2	1,9	1,6	2,3
*Dauer in der Notaufnahme in % (n* *=* *354.547; 99,5* *%)*										
Bis 1 h		18,2	15,6	13,3	10,6	8,4	6,9	5,9	4,9	12,0
> 1 bis 2 h		26,5	24,1	22,4	20,1	17,3	15,7	14,9	16,2	20,8
> 2 bis 3 h		22,1	21,8	21,8	21,6	21,1	21,0	20,9	21,4	21,6
> 3 bis 4 h		13,7	14,9	15,6	16,6	17,7	18,3	19,3	20,4	16,3
> 4 h		19,5	23,6	26,8	31,1	35,5	38,1	39,0	37,0	29,4
*Verlegungs‑/Entlassungsart in % (n* *=* *279.101; 78,3* *%)*										
Ambulant		81,3	75,8	67,4	56,0	43,2	36,9	35,6	43,5	58,7
Stationär (peripher)		16,1	20,4	26,2	34,2	44,0	49,5	52,5	48,5	33,1
Stationär (intensiv)		2,1	3,1	5,6	8,9	11,9	12,5	10,9	7,2	7,4
Verlegung (extern)		0,5	0,7	0,8	0,9	1,0	1,0	1,0	0,8	0,8

^a^Informationen nur aus 8 Kliniken verfügbar, deren Anteil gültiger Werte: 90,4 %

In Zusammenschau aller Ergebnisse gab es kaum geschlechterspezifische Unterschiede (eTabellen 4‑6).

## Diskussion

In der Analyse von fast 360.000 Besuchen aus 11 deutschen Notaufnahmen zeigte sich, dass 4 von 10 erwachsenen Notfallpatienten 65+ Jahre alt waren. Von diesen wurden beinahe zwei Drittel mit einem Transportmittel der Notfallversorgung eingeliefert und ebenso zwei Drittel als dringlich ersteingeschätzt, was einem deutlich höheren Anteil als bei Jüngeren entspricht. Internistische Krankheitsbilder waren in Notaufnahmen die häufigsten Gründe, sie kamen im Vergleich zu Jüngeren bei Älteren fast doppelt so häufig vor. Zudem wurden ältere Notfallpatienten deutlich häufiger stationär aufgenommen.

### Anteil älterer Notaufnahmepatienten

Ähnlich zu Ergebnissen von Wallstab et al. [26] liegt in unserer Studie der Anteil älterer Notfallpatienten mit knapp 40 % höher als in weiteren Studien und scheint damit einem generellen Trend zu folgen. Noch vor fast 20 Jahren beschrieben Aminzadeh et al. in einem Übersichtsartikel einen Anteil von bis zu 21 % im angloamerikanischen Raum [27]. Pines et al. konnten in den USA von 2001 zu 2009 bereits einen deutlichen Anstieg von Notaufnahmebesuchen bei Älteren verzeichnen [28]. Auch im deutschsprachigen Raum stieg dieser über die letzten Jahre, wie eine Studie aus 2013 aufzeigt [29]. In einer aktuellen Studie von 2020 waren 27,4 % 65+ Jahre alt [6]. Der im Literaturvergleich höhere Anteil lässt sich, neben unserer Aktualität, auch teilweise durch den Ausschluss von Kindern erklären.

Im Zuge des demografischen Wandels ist die Anzahl Pflegebedürftiger über die letzten Jahre deutlich gestiegen [30], was eine Zunahme älterer Patienten in Notaufnahmen zu Teilen erklärt. Gleichzeitig spielen Versorgungsdefizite im ambulanten Sektor eine Rolle [7]. Durch den zunehmenden Hausärztemangel, speziell im ländlichen Bereich, wird die Versorgung insbesondere für Ältere oder Pflegebedürftige erschwert, da gleichzeitig in den vergangenen Jahren die Anzahl an Hausbesuchen deutlich zurück ging [31]. Diese Klientel ist häufig in ihrer Mobilität und Selbstständigkeit eingeschränkt, lebt allein oder in Pflegeeinrichtungen. Gerade für Pflegeheimbewohner werden Defizite in der Notfallversorgung diskutiert, sodass oft der Rettungsdienst anstatt des Hausarztes gerufen wird [32], was wiederum zu mehr Notaufnahmekonsultationen führen kann.

### Zuweiser/Transport

Von allen Notaufnahmepatienten waren 45 % Selbstvorsteller. Während sich die jüngeren Patienten mit 58,3 % selbst in der Notaufnahme vorstellten, war dies bei den älteren nur bei 24,2 % der Fall. Dabei wurden Ältere im Vergleich zu Jüngeren deutlich häufiger mit einem Transportmittel (60,1 % vs. 25,9 %) und vorrangig mit dem Rettungsdienst zugewiesen (34,3 % vs. 15,4 %). Damit sind unsere Befunde mit denen der Literatur vergleichbar [33].

Auffällig und damit diskussionswürdig ist unser Anteil von knapp 70 % aller Notfallpatienten, die zu klassischen Praxissprechzeiten (7.00 bis 19.00 Uhr) kommen. Hierbei gab es keine deutlichen altersspezifischen Unterschiede. Identische Ergebnisse wurden auch im Krankenhausreport von 2018 beschrieben [34]. Ein Grund dafür scheint die Unkenntnis über den ärztlichen Bereitschaftsdienst zu sein [3, 35]. Darüber hinaus können Patienten häufig nicht einschätzen, ob eine spätere Behandlung durch den Bereitschaftsdienst ausreichend ist [2, 3]. Gleichzeitig würden fast zwei Drittel Notfallstrukturen der KV nutzen, wären sie bekannt oder vorhanden [14]. Nicht oder spät verfügbare Haus- oder Facharzttermine waren ein weiterer Beweggrund. Überwiegend für Jüngere gelten allerdings als Hauptmotive ständige Verfügbarkeit, Zeitautonomie sowie die sofortige Verfügbarkeit umfangreicher Diagnostik [12]. Letzteres wird aber auch nicht selten von niedergelassenen Ärzten genutzt, da gerade Ältere häufig atypische Beschwerden aufweisen und Laborergebnisse in Notaufnahmen zeitnah zur Verfügung stehen. Darüber hinaus stellen insbesondere für ältere oder immobile Patienten regional unterschiedliche Organisationen des kassenärztlichen Bereitschaftsdienstes ohne feste Notdienstpraxen und -zeiten Barrieren dar [36]. Möglicherweise wird dann auf den Rettungsdienst zurückgegriffen, was den höheren Anteil dieser Klientel erklären könnte [34]. Die im Krankenhausstrukturgesetz 2015 angedachten integrierten Notfallzentren (INZ) mit einer gemeinsamen Versorgung von Notfallpatienten durch die kassenärztliche Vereinigung und das Krankenhaus sowie unter einer entsprechenden Steuerung könnten nach ersten Modellprojekten wie in Frankfurt Höchst eine Alternative insbesondere für mobile und jüngere Patienten sein. Sie konnten nachweislich dem unkontrollierten Patientenzugang in Notaufnahmen entgegenwirken und dadurch einen 30%igen Rückgang von ambulanten Notaufnahmevorstellungen verzeichnen, da hauptsächlich fußläufige Patienten an einer zentralen Anlaufstelle in die richtige Versorgungsebene vermittelt wurden [37]. Für immobile, mit Rettungsmitteln transportierte ältere Menschen stellen INZ allerdings keine Alternative dar.

### Prozesszeiten

Ca. 30 % aller Patienten verblieben über 4 h in der Notaufnahme, wobei dieser Anteil mit zunehmendem Alter deutlich anstieg. Otto et al. konnten in ihrer Studie aus 2022 eine durchschnittliche Aufenthaltsdauer aller Notfallpatienten von 208 min ermitteln [38], was sich mit unseren Ergebnissen deckt. Darüber hinaus zeigten sie auf, dass stationär aufgenommene Patienten 64 min länger in der Notaufnahme verweilten, als ambulant verbliebene, unabhängig von anderen Variablen [38].

Andere Studien von Rygiel et al. [6] sowie Biber et al. [39] zeigten zwar durchschnittlich geringere Aufenthaltsdauern, allerdings handelt es sich um monozentrische Studien mit geringerer Patientenzahl. Letztere Studie konnte allerdings ebenfalls eine stärkere Korrelation zwischen dem Alter und der Aufenthaltsdauer bei traumatologischen Patienten aufzeigen [39]. Perdahl et al. zeigten 2017 in einer schwedischen Kohortenstudie, dass die mit langen Aufenthaltsdauern assoziierten patientenbezogenen Faktoren u. a. das weibliche Geschlecht und Alter 65+ Jahre waren [40].

Insgesamt spiegelt dies wider, dass die Länge der Prozesszeiten multifaktoriell beeinflusst wird und u. a. von demografischen und gesundheitlichen, organisatorischen als auch strukturellen Faktoren abhängt [22, 38]. Trotzdem zeigt sich auch dabei die besondere Bedeutung des Faktors Alter.

### Stationäre Aufnahme vs. ambulante Behandlung

Der Anteil ambulanter Notaufnahmekontakte lag in unserer Studie bei knapp 59 % mit deutlichen Altersunterschieden. Während die Mehrheit der Jüngeren ambulant versorgt wurde, stieg mit dem Alter die Wahrscheinlichkeit einer stationären Aufnahme.

Knapp 30 % aller Krankheitsbilder waren aus dem internistischen Fachbereich. Während bei den Jüngeren mit 30,8 % die Verletzungen und Intoxikationen führen, waren es bei den Älteren mit 38,6 % die internistischen Krankheitsbilder, was sich gerade bei den älteren Patienten in anderen Studien uneinheitlich darstellt [5, 20, 36].

Hinsichtlich des ambulanten Anteils, besonders für Jüngere und eigentlich Gesunde sowie die urbane Bevölkerung, bestätigt der Literaturvergleich zum Teil unsere Daten und zeigt auf, dass diese möglicherweise auch im ambulanten Sektor hätten behandelt werden könnten [20, 29, 33]. Ein Gutachten der MCK/DGINA aus 2015 ermittelte, das ein Drittel in niedergelassenen Praxen versorgt hätte werden können [41].

Ähnlich zu unseren Ergebnissen beschrieben Singal et al. 1992 in einer US-amerikanischen Studie eine doppelt so hohe stationäre Aufnahmerate bei älteren Notfallpatienten [42]. Insbesondere bei Krankenhaustransporten von Pflegebedürftigen und Heimbewohnern belegten Kada et al., dass knapp ein Drittel kurzer stationärer Aufenthalte (nicht länger als 2 Tage) und fast 40 % der ambulanten Behandlungen in Notaufnahmen sich als im medizinischen Sinne vermeidbar erwiesen [43]. Der Rückgang an Hausbesuchen könnte auch hier eine große Rolle spielen, vor allem bei den älteren sowie pflegebedürftigen Patienten. Nicht selten ist die „soziale Indikation“ und damit, ob der ältere Patient trotz vorhandener ambulanter Strukturen auch tatsächlich im häuslichen Umfeld adäquat versorgt werden kann, mit ausschlaggebend. Mit dem Wissen, dass ältere vulnerablere Patienten gehäuft bei Notaufnahmevorstellungen oder sogar stationären Aufnahmen in der Folge unter Allgemeinzustandsverschlechterung, Autonomieverlust und einer erhöhten Mortalität leiden [8], bedarf es einer Debatte zur zukünftigen Versorgung dieser Klientel, wie sie bereits von unterschiedlichen Institutionen gefordert wurde [2, 4, 34, 44].

### Stärken und Schwächen

Wesentliche Stärke unserer Studie ist die hohe Fallzahl aus mehreren deutschlandweit verteilten Notaufnahmen mit unterschiedlichen Versorgungsstufen, was das Potenzial für Verzerrungen minimiert. Hauptlimitationen sind die Anzahl fehlender Werte bei einzelnen Variablen (z. B. Ausschluss von 3 Zentren, 35,7 % bei der Dauer bis zum ersten Arztkontakt und 21,7 % bei der Verlegungs‑/Entlassungsart) sowie 3 aufgrund von unvollständigen Datensätzen komplett ausgeschlossenen Kliniken (nähere Ausführungen siehe eBox).

## Schlussfolgerung

Ältere Notaufnahmepatienten scheinen die „klassischen Notfallpatienten“ zu sein. Diese Patientenklientel weist die akuteren Krankheitsbilder, höhere Dringlichkeit, häufige rettungsdienstbegleitete Transporte, eine längere Aufenthaltsdauer in der Notaufnahme und höhere stationäre Aufnahmequoten auf. Die Daten in Deutschland passen zu internationalen Zahlen. Sie untermauern die zunehmend lauter werdende Forderung, Notaufnahmen in ihren Ausstattungen und Prozessen zunehmend an diese älteren und hochbetagten Patienten anzupassen.

## Kernaussagen

- In einer Analyse von ca. 360.000 Besuchen aus 11 Notaufnahmen waren 4 von 10 erwachsenen Notaufnahmepatienten 65+ Jahre alt.
- Ältere Notfallpatienten werden häufiger rettungsdienstbegleitetet vorgestellt und weisen die akuteren Krankheitsbilder sowie eine dringlichere Ersteinschätzung auf.
- Internistische Krankheitsbilder waren in Notaufnahmen die häufigsten, sie kamen im Vergleich zu Jüngeren bei Älteren fast doppelt so häufig vor.
- Eine stationäre Aufnahme bei älteren ist im Vergleich zu jüngeren Notfallpatienten häufiger, was zusammenfassend aufzeigt, dass sie die klassischen Notfallpatienten sind.
- In Zusammenschau dieser Erkenntnisse sollte in Notaufnahmen eine Optimierung der Prozesse und Strukturen auf ältere Patienten erfolgen.

### Supplementary Information





## References

[1] Ministerium für Soziales, Gesundheit und Gleichstellung (2021) Wahlperiode NL–18.: Bericht der Enquetekommission „Sicherstellung der ambulanten und stationären medizinischen Versorgung in Niedersachsen – für eine qualitativ hochwertige und wohnortnahe medizinische Versorgung“. https://www.ms.niedersachsen.de. Zugegriffen: 11. Okt. 2021

[2] Gutachten 2018 – SVR Gesundheit. https://www.svr-gesundheit.de/gutachten/gutachten-2018/. Zugegriffen: 18. Okt. 2021

[3] Köster C, Wrede S, Herrmann T, Meyer S, Willms G, Broge B, Szecsenyi J (2016). Ambulante Notfallversorgung. Analyse und Handlungsempfehlungen. Institut, Ambulante Notfallversorgung Analyse und Handlungsempfehlungen.

[4] (2020) Stellungnahme des Marburger Bund Bundesverbandes zu dem Referentenentwurf des Bundesministeriums für Gesundheit, Entwurf eines Gesetzes zur Reform der Notfallversorgung. https://www.marburger-bund.de/bundesverband/der-marburger-bund/stellungnahmen. Zugegriffen: 19. Okt. 2021

[5] Mangiapane S, von Czihal TSD (2021) Entwicklung der ambulanten Notfallversorgung in Deutschland von 2009 bis 2020. https://www.zi.de. Zugegriffen: 23. Okt. 2021

[6] Rygiel K, Fimmers R, Schacher S, Dormann H, Gräff I (2020). Older emergency patients in the emergency department: a key performance indicator analysis based on the DIVI emergency department protocol. Med Klin Intensivmed Notfallmed.

[7] Groening M, Grossmann F, Hilmer T, Singler K, Somasundaram R, Wilke P (2017). Ältere Notfallpatienten – Blickschärfung notwendig. Dtsch Arztebl.

[8] Singler K, Dormann H, Dodt C (2016). Der geriatrische Patient in der Notaufnahme: Positionspapier der Deutschen Gesellschaft interdisziplinäre Notfall- und Akutmedizin (DGINA), der Deutschen Gesellschaft für Geriatrie (DGG), der Deutschen Gesellschaft für Gerontologie und Geriatrie (DGGG. Notfall Rettungsmed.

[9] Groening M, Wilke P (2020). Medizinische Klinik – Intensivmedizin und Notfallmedizin.

[10] Singler K, Heppner HJ (2021). Why geriatric expertise is needed in emergency medicine. Z Gerontol Geriatr.

[11] Mooijaart SP (2021). Improving the care for older emergency department patients: the Acutely Presenting Older Patient study. Z Gerontol Geriat.

[12] Schmiedhofer MH, Searle J, Slagman A, Möckel M (2017). Inanspruchnahme zentraler Notaufnahmen: Qualitative Erhebung der Motivation von Patientinnen und Patienten mit nichtdringlichem Behandlungsbedarf. Gesundheitswesen.

[13] Reinhold AK, Greiner F, Schirrmeister W, Walcher F, Erdmann B (2021). Even low-acuity patients prefer hospital-based emergency care: a survey of non-urgent patients in an emergency department with unique regional position. Med Klin Intensivmed Notfallmed.

[14] Somasundaram R, Geissler A, Leidel B, Wrede C (2018). Beweggründe für die Inanspruchnahme von Notaufnahmen – Ergebnisse einer Patientenbefragung. Gesundheitswesen.

[15] PM: Demografischer Wandel in Deutschland: „Wir brauchen mehr lokale Daten zur Versorgung alter Menschen“. https://www.dggeriatrie.de/presse/pressemeldungen/1490-pm-demografischer-wandel-in-deutschland-„wir-brauchen-mehr-lokale-daten-zur-versorgung-alter-menschen“. Zugegriffen: 23. Okt. 2021

[16] Rauch J, Denter M, Hübner U (2019). Use of emergency departments by frail elderly patients: temporal patterns and case complexity. Studies in health technology and Informatics.

[17] Schöpke T, Plappert T (2011). Kennzahlen von Notaufnahmen in Deutschland. Notfall Rettungsmed.

[18] Klauber J, Geraedts M (2019). Krankenhaus-Report 2019.

[19] Statistisches Bundesamt (2019) Ergebnisse der 14. koordinierten Bevölkerungsvorausberechnung – Statistisches Bundesamt. Statistisches Bundesamt. https://www.destatis.de/DE/Themen/Gesellschaft-Umwelt/Bevoelkerung/Bevoelkerungsvorausberechnung/Tabellen/variante-1-2-3-altersgruppen.html. Zugegriffen: 31. Jan. 2021

[20] Wahlster P, Czihal T, Gibis B, Henschke C (2020). Developments in emergency care—analysis of emergency cases in in- and outpatient care from 2009 to 2015 in Germany. Gesundheitswesen.

[21] Trentzsch H, Dodt C, Gehring C, Veser A, Jauch KW, Prückner S (2020). Analysis of treatment figures in the munich emergency rooms 2013–2014. Gesundheitswesen.

[22] Brammen D, Greiner F, Kulla M (2022). Das AKTIN-Notaufnahmeregister – kontinuierlich aktuelle Daten aus der Akutmedizin. Med Klin Intensivmed Notfallmed.

[23] Gemeinsamer Bundesausschuss (2018). Gestuftes System von Notfallstrukturen in Krankenhäusern.

[24] Marsden J, Windle J, Mackway-Jones K (2013). Emergency triage. Emergency nurse: The journal of the RCN Accident and Emergency Nursing. Association.

[25] Ramroth H, Specht-Leible N, Brenner H (2005). Hospitalisations before and after nursing home admission: A retrospective cohort study from Germany [1]. Age Ageing.

[26] Wallstab F, Greiner F, Schirrmeister W (2022). German emergency department measures in 2018: a status quo based on the Utstein reporting standard. BMC Emerg Med.

[27] Aminzadeh F, Dalziel WB (2002). Older adults in the emergency department: a systematic review of patterns of use, adverse outcomes, and effectiveness of interventions. Ann Emerg Med.

[28] Pines JM, Mullins PM, Cooper JK, Feng LB, Roth KE (2013). National trends in emergency department use, care patterns, and quality of care of older adults in the United States. J Am Geriatr Soc.

[29] Vilpert S (2013) Konsultationen in Schweizer Notfallstationen | OBSAN. https://www.obsan.admin.ch/de/publikationen/konsultationen-schweizer-notfallstationen. Zugegriffen: 27. Okt. 2021

[30] Böhm K (2021) Statistik Campus – Datenreport – Statistisches Bundesamt. https://www.destatis.de/DE/Service/Statistik-Campus/Datenreport/_inhalt.html. Zugegriffen: 29. Okt. 2021

[31] (2018) Ärzte machen weniger Hausbesuche. Deutsche Ärzteblatt. https://www.aerzteblatt.de/nachrichten/95839/Aerzte-machen-weniger-Hausbesuche. Zugegriffen: 1. Nov. 2021

[32] Arendts G, Howard K (2010). The interface between residential aged care and the emergency department: a systematic review. Age Ageing.

[33] Schöpke T, Dodt C, Brachmann M, Schnieder W, Petersen PF, Böer J (2014). Status report from German emergency departments: Results from the DGINA member questionnaire 2013. Notfall und Rettungsmedizin.

[34] Slowik M, Wehner C, Dräther H, Fahlenbrach C, Richard S (2018) Krankenhausreport 2018 kap. 13, Sektorübergreifende Neuordnung der Notfallversorgung. https://www.wido.de. Zugegriffen: 4. Nov. 2021

[35] Kassenärztliche Bundesvereinigung (2020). Versichertenbefragung der Kassenärztlichen Bundesvereinigung 2020.

[36] Slowik M, Bockhorst K (2020). Reform der Notfallversorgung. Qualitätsmonitor 2020.

[37] (2019) Notfallversorgung: Wege zu mehr Patientensteuerung. Ärzteblatt. https://www.aerzteblatt.de/archiv. Zugegriffen: 23. Okt. 2021

[38] Otto R, Blaschke S, Schirrmeister W, Drynda S, Walcher F, Greiner F (2022). Length of stay as quality indicator in emergency departments: analysis of determinants in the German Emergency Department Data Registry (AKTIN registry). Intern Emerg Med.

[39] Biber R, Bail HJ, Sieber C, Weis P, Christ M, Singler K (2012). Correlation between age, emergency department length of stay and hospital admission rate in emergency department patients aged ≥70 years. Gerontology.

[40] Perdahl T, Axelsson S, Svensson P, Djärv T (2017). Patient and organizational characteristics predict a long length of stay in the emergency department—a Swedish cohort study. Eur J Emerg Med.

[41] Neubauer C, Minartz C, Niedermeier G (2016). Kritische Analyse des „Gutachtens zur ambulanten Notfallversorgung im Krankenhaus-Fallkostenkalkulation und Strukturanalyse“ der MCK in Kooperation mit der DGINA vom 17.02.2015 Expertise für das Zentralinstitut für die kassenärztliche Versorgung (Zi).

[42] Singal BM, Hedges JR, Rousseau EW (1992). Geriatric patient emergency visits part I: Comparison of visits by geriatric and younger patients. Ann Emerg Med.

[43] Kada O, Brunner E, Likar R (2011). Vom Pflegeheim ins Krankenhaus und wieder zurück... Eine multimethodale Analyse von Krankenhaustransporten aus Alten- und Pflegeheimen. Z Evid Fortbild Qual Gesundhwes.

[44] Gutachten 2021 – SVR Gesundheit. https://www.svr-gesundheit.de/gutachten/gutachten-2021/. Zugegriffen: 4. Nov. 2021

