# Supplementary material for: Characteristics of older versus younger emergency patients: Analysis of over 356,000 visits from the AKTIN German emergency department data registry
Source: Med Klin Intensivmed Notfmed. 2022 Nov 4;119(1):18–26. [Article in German] doi: 10.1007/s00063-022-00968-8 (PMC10803396; doi:10.1007/s00063-022-00968-8)
Supplement: Supplementary file 1 [file 63_2022_968_MOESM1_ESM.docx]

eTabelle 1: Charakteristika der eingeschlossenen AKTIN-Notaufnahmen

| **Krankenhaus bzw. Notaufnahme** | **Anzahl Betten** | **Versorgungsstufe gemäß GBA-Beschluss** | **Stufe (GBA)** |
| --- | --- | --- | --- |
| Paracelsus-Klinik Henstedt-Ulzburg | 184 | Basisnotfallversorgung | 1 |
| Ostalb-Klinikum Aalen | 400 | Erweiterte Notfallversorgung | 2 |
| Pius-Hospital Oldenburg | 408 | Erweiterte Notfallversorgung | 2 |
| Evangelisches Krankenhaus Oldenburg | 420 | Erweiterte Notfallversorgung | 2 |
| Klinikum Memmingen | 500 | Umfassende Notfallversorgung | 3 |
| Klinikum Wolfsburg | 547 | Umfassende Notfallversorgung | 3 |
| Klinikum Fürth | 765 | Erweiterte Notfallversorgung | 2 |
| Klinikum Aschaffenburg-Alzenau | 785 | Erweiterte Notfallversorgung | 2 |
| Universitätsmedizin Göttingen (INA) | 1.440 | Umfassende Notfallversorgung | 3 |
| Universitätsklinikum Augsburg | 1.740 | Umfassende Notfallversorgung | 3 |
| Klinikum Chemnitz | 1.765 | Umfassende Notfallversorgung | 3 |
| ***GESAMT*** | ***8.954*** |  |  |

eTabelle 2: Charakteristika von Notaufnahmebesuchen nach Altersgruppen (in %)

| **Variablen mit einzelnen Ausprägungen** | **18 - 64 Jahre** | **65+ Jahre** |
| --- | --- | --- |
| **Fallzahlen (n = 356.354; 100%)** | **216.894 (60,9%)** | **139.460 (39,1%)** |
| Frauen | 100.827 (46,5%) | 74.191 (53,2%) |
| Männer | 116.067 (53,5%) | 65.269 (46,8%) |
| **Zu-/Einweiser in % (n = 346.202; 97,2%)** | | |
| Zuweisung nicht durch Arzt | 58,3 | 24,2 |
| Rettungsdienst | 15,4 | 34,3 |
| Vertragsarzt / Praxis | 12,8 | 20,4 |
| Notarzt | 6,4 | 12,2 |
| KV-Notdienst + KV-Notfallpraxis | 3,9 | 3,9 |
| Klinikverlegung + andere | 3,3 | 5,0 |
| **Transportart in % (n = 350.236; 98,3%)** | | |
| Ohne | 74,1 | 39,9 |
| RTW | 18,4 | 41,8 |
| NAW / NEF / ITW | 5,6 | 10,4 |
| KTW | 1,6 | 7,5 |
| RTH / ITH | 0,4 | 0,4 |
| **Ersteinschätzung in % (n = 338.984; 95,1%)** | | |
| Stufe 1 - rot | 0,9 | 1,8 |
| Stufe 2 - orange | 12,9 | 21,8 |
| Stufe 3 - gelb | 33,3 | 42,5 |
| Stufe 4 - grün | 47,0 | 31,1 |
| Stufe 5 - blau | 5,9 | 2,8 |
| **Uhrzeit in % (n = 356.354; 100%)** | | |
| 00:00 bis 07:59 | 13,1 | 11,4 |
| 08:00 bis 19:59 | 69,8 | 76,7 |
| 20:00 bis 23:59 | 17,1 | 11,9 |
| **Wochentag (n = 356.354; 100%)** | | |
| Montag | 15,5 | 16,3 |
| Dienstag | 14,4 | 15,1 |
| Mittwoch | 13,9 | 14,5 |
| Donnerstag | 13,7 | 14,9 |
| Freitag | 14,7 | 15,0 |
| Sonnabend | 14,0 | 12,5 |
| Sonntag | 13,8 | 11,7 |
| **Dauer bis zum ersten Arztkontakt in % (n = 229.219 *)** | | |
| bis 1 Std. | 69,4 | 73,1 |
| > 1 bis 2 Std. | 21,4 | 18,8 |
| > 2 bis 3 Std. | 6,8 | 6,0 |
| > 3 bis 4 Std. | 2,3 | 2,2 |
| **Dauer in der Notaufnahme in % ( n = 354.547; 99,5%)** | | |
| bis 1 Std. | 15,1 | 7,1 |
| > 1 bis 2 Std. | 23,8 | 16,0 |
| > 2 bis 3 Std. | 21,9 | 21,0 |
| > 3 bis 4 Std. | 14,9 | 18,4 |
| > 4 Std. | 24,3 | 37,5 |
| **Verlegung/Entlassung in % (n = 279.101; 78,3%)** | | |
| ambulant | 71,8 | 38,7 |
| stationär (peripher) | 23,0 | 48,4 |
| stationär (intensiv) | 4,5 | 11,9 |
| Verlegung (extern) | 0,7 | 1,0 |

* Informationen nur aus 8 Kliniken verfügbar, deren Anteil gültiger Werte: 90,4%

eTabelle 3: Diagnosen von 300.127 Notaufnahmebesuchen nach Altersgruppen (in %)

| **Altersgruppe** | **18 – 64 Jahre** | **65+ Jahre** |
| --- | --- | --- |
| **336.455 Notaufnahmediagnosen *, ICD 10 Gruppe** | **193.024** | **143.431** |
| **Internistische Erkrankungen** | **22,5** | **38,8** |
| Kardiovaskuläre Erkrankungen | 5,7 | 14,8 |
| Infektionen | 6,0 | 8,3 |
| Gastrointestinale Erkrankungen | 6,2 | 6,5 |
| Endokrine, Ernährungs- und Stoffwechselerkrankungen | 1,5 | 4,8 |
| Respiratorische Erkrankungen | 2,2 | 2,8 |
| Neubildungen | 0,9 | 1,7 |
| **Andere Erkrankungen **** | **30,6** | **25,7** |
| **Verletzungen und Intoxikation** | **30,9** | **21,1** |
| R55 Synkope und Kollaps | 1,1 | 1,9 |
| S00-S09 Kopfes | 4,5 | 5,1 |
| S10-S19 Halses | 1,2 | 0,3 |
| S20-S29 Thorax | 1,5 | 1,4 |
| S30-S39 Abdomen, Lumbosakralgegend, Lendenwirbelsäule, Becken | 0,9 | 1,4 |
| S40-S49 Schulter und Oberarm | 1,3 | 1,2 |
| S50-S59 Ellenbogen und Unterarm | 1,4 | 1,0 |
| S60-S69 Handgelenk und Hand | 5,8 | 1,1 |
| S70-S79 Hüfte und Oberschenkel | 0,6 | 2,2 |
| S80-S89 Knie und Unterschenkel | 2,5 | 1,2 |
| S90-S99 Knöchelregion und Fuß | 3,8 | 0,5 |
| T00-T07 mit Beteiligung mehrerer Körperregionen | 0,4 | 0,2 |
| T08-T14 nicht näher bezeichneter Rumpfteile, der Extremitäten oder anderer Körperregionen | 1,6 | 0,7 |
| T15-T19 Folgen des Eindringens eines Fremdkörpers durch eine natürliche Körperöffnung | 0,6 | 0,2 |
| T20-T32 Verbrennungen oder Verätzungen | 0,4 | 0,1 |
| T33-T35 Erfrierungen | 0,0 | 0,0 |
| T36-T50 Vergiftungen durch Arzneimittel, Drogen und biologisch aktive Substanzen | 0,2 | 0,1 |
| T51-T65 Toxische Wirkungen von vorwiegend nicht medizinisch verwendeten Substanzen | 0,8 | 0,1 |
| T66-T78 Sonstige und nicht näher bezeichnete Schäden durch äußere Ursachen | 0,9 | 0,3 |
| T79-T79 Bestimmte Frühkomplikationen eines Traumas | 0,1 | 0,1 |
| T80-T88 Komplikationen bei chirurgischen Eingriffen, medizin. Behandlung, anderenorts n. k. | 1,0 | 2,0 |
| T89-T89 Sonstige Komplikationen eines Traumas, anderenorts nicht klassifiziert | 0,2 | 0,1 |
| T90-T98 Folgen von Verletzungen, Vergiftungen + sonstigen Auswirkungen äußerer Ursachen | 0,1 | 0,0 |
| **Muskuloskeletalle Erkrankungen** | **7,0** | **4,7** |
| **Cerebrovaskuläre Erkrankungen** | **1,8** | **6,2** |
| **Erkrankungen des Nervensystems und Sinnesorgane** | **3,5** | **2,3** |
| **Psychische und Verhaltensstörungen** | **3,7** | **1,2** |

ICD = International Classification of Diseases, Gruppierung nach Ramroth

* mehrere Diagnosen pro Fall möglich

** detaillierte Aufschlüsselung siehe Tabelle 2

eTabelle 4: Basischarakter von Notaufnahmebesuchen nach Alter und Geschlecht (in %)

| **n = 356.354** | **18-34** | | **35-44** | | **45-54** | | **55-64** | | **65-74** | | **75-84** | | **85-94** | | **95+** | | **Gesamt** | |
| --- | --- | --- | --- | --- | --- | --- | --- | --- | --- | --- | --- | --- | --- | --- | --- | --- | --- | --- |
|  | **F** | **M** | **F** | **M** | **F** | **M** | **F** | **M** | **F** | **M** | **F** | **M** | **F** | **M** | **F** | **M** | **F** | **M** |
| **Zu-/Einweiser in % (n = 346.202)** | |  |  |  |  |  |  |  |  |  |  |  |  |  |  |  |  |  |
| Zuweisung nicht durch Arzt | 66,8 | 67,1 | 62,1 | 62,4 | 54,6 | 52,8 | 46,0 | 42,0 | 34,2 | 31,9 | 23,3 | 23,1 | 13,7 | 16,3 | 9,6 | 13,1 | **44,1** | **45,8** |
| Rettungsdienst | 11,6 | 14,9 | 12,1 | 14,7 | 14,5 | 17,6 | 18,2 | 21,2 | 24,9 | 25,9 | 34,6 | 34,7 | 45,6 | 43,1 | 55,2 | 53,3 | **22,8** | **22,7** |
| Vertragsarzt / Praxis | 10,7 | 7,2 | 13,1 | 10,3 | 16,3 | 13,5 | 19,4 | 18,3 | 22,1 | 20,9 | 21,0 | 21,1 | 17,5 | 18,7 | 14,9 | 15,4 | **16,7** | **14,8** |
| Notarzt | 3,8 | 5,2 | 4,9 | 6,2 | 6,4 | 8,7 | 8,2 | 10,2 | 10,1 | 12,1 | 12,3 | 11,9 | 14,3 | 13,2 | 13,7 | 11,5 | **8,3** | **9,0** |
| KV-Notdienst / -Notfallpraxis | 3,9 | 3,5 | 4,3 | 3,8 | 4,3 | 3,7 | 4,0 | 3,8 | 3,9 | 3,9 | 3,8 | 3,8 | 4,2 | 3,9 | 3,7 | 4,6 | **4,0** | **3,8** |
| Klinikverlegung + andere | 3,1 | 2,1 | 3,5 | 2,6 | 3,9 | 3,6 | 4,1 | 4,5 | 4,8 | 5,3 | 4,9 | 5,4 | 4,6 | 4,8 | 2,9 | 2,1 | **4,1** | **3,8** |
| **Transportart in % (n = 350.236)** | |  |  |  |  |  |  |  |  |  |  |  |  |  |  |  |  |  |
| ohne | 82,1 | 77,3 | 79,8 | 75,5 | 74,6 | 68,9 | 67,0 | 61,3 | 55,0 | 51,4 | 39,3 | 39,8 | 22,4 | 26,2 | 14,2 | 16,4 | **60,7** | **60,7** |
| RTW | 13,6 | 17,0 | 14,7 | 17,3 | 17,8 | 21,2 | 22,7 | 26,0 | 31,2 | 32,2 | 42,5 | 41,4 | 55,1 | 52,2 | 63,1 | 61,7 | **27,8** | **27,3** |
| NAW / NEF / ITW | 3,2 | 4,4 | 4,1 | 5,6 | 5,4 | 7,5 | 7,1 | 9,2 | 8,8 | 10,9 | 10,3 | 10,6 | 11,3 | 10,6 | 10,8 | 9,8 | **7,0** | **7,9** |
| KTW | 0,8 | 1,0 | 1,1 | 1,2 | 1,9 | 2,0 | 2,9 | 3,0 | 4,6 | 5,2 | 7,5 | 7,9 | 10,8 | 10,6 | 11,7 | 11,7 | **4,1** | **3,8** |
| RTH / ITH + andere | 0,2 | 0,3 | 0,3 | 0,4 | 0,3 | 0,4 | 0,3 | 0,5 | 0,4 | 0,4 | 0,4 | 0,3 | 0,3 | 0,3 | 0,3 | 0,5 | **0,3** | **0,4** |
| **Ersteinschätzung in % (n = 338.984)** | |  |  |  |  |  |  |  |  |  |  |  |  |  |  |  |  |  |
| Stufe 1 - rot | 0,4 | 0,8 | 0,4 | 0,6 | 0,8 | 1,1 | 1,1 | 2,0 | 1,6 | 2,3 | 1,7 | 1,9 | 1,7 | 1,7 | 1,3 | 1,1 | **1,1** | **1,4** |
| Stufe 2 - orange | 8,6 | 9,6 | 11,0 | 13,4 | 13,1 | 16,6 | 15,9 | 19,8 | 20,1 | 22,7 | 21,4 | 22,1 | 22,5 | 21,7 | 24,1 | 21,2 | **15,7** | **17,0** |
| Stufe 3 - gelb | 33,5 | 26,8 | 35,5 | 30,7 | 36,2 | 34,0 | 37,9 | 38,1 | 41,6 | 42,3 | 42,3 | 43,8 | 42,1 | 43,7 | 40,0 | 41,1 | **38,1** | **35,8** |
| Stufe 4 - grün | 49,8 | 56,3 | 46,4 | 49,3 | 44,3 | 43,3 | 40,5 | 36,2 | 33,2 | 29,6 | 31,8 | 29,6 | 31,7 | 30,6 | 33,0 | 35,1 | **40,3** | **41,3** |
| Stufe 5 - blau | 7,7 | 6,5 | 6,6 | 6,0 | 5,7 | 5,0 | 4,6 | 3,9 | 3,6 | 3,2 | 2,8 | 2,6 | 2,0 | 2,3 | 1,6 | 1,5 | **4,9** | **4,5** |

F = Frauen, M = Männer

eTabelle 5: Diagnosen von 300.127 Notaufnahmebesuchen nach Alter und Geschlecht (in %)

| **ICD Gruppe**  **336.455 NA-Diagnose *** | **18-34** | | **35-44** | | **45-54** | | **55-64** | | **65-74** | | **75-84** | | **85-94** | | **95+** | | **gesamt** | |
| --- | --- | --- | --- | --- | --- | --- | --- | --- | --- | --- | --- | --- | --- | --- | --- | --- | --- | --- |
|  | **F** | **M** | **F** | **M** | **F** | **M** | **F** | **M** | **F** | **M** | **F** | **M** | **F** | **M** | **F** | **M** | **F** | **M** |
| n je Alter | 35.970 | 37.112 | 16.316 | 18.850 | 17.954 | 21.747 | 19.924 | 25.151 | 20.277 | 23.597 | 34.399 | 30.432 | 19.961 | 12.102 | 2.007 | 656 | 166.808 | 169.647 |
| Internistische Erkrankungen | 18,6 | 16,1 | 19,7 | 19,5 | 24,4 | 25,3 | 29,8 | 32,0 | 36,4 | 39,6 | 38,0 | 40,3 | 39,3 | 39,6 | 34,7 | 34,5 | **29,5** | **29,4** |
| Kardiovaskuläre Erkrankungen | 2,4 | 1,9 | 3,8 | 4,3 | 6,9 | 8,0 | 10,0 | 11,8 | 14,1 | 14,4 | 15,5 | 15,3 | 15,1 | 13,6 | 10,8 | 10,4 | **9,7** | **9,4** |
| Infektionen | 7,2 | 5,5 | 5,9 | 5,5 | 5,5 | 5,7 | 6,2 | 6,1 | 6,9 | 8,3 | 7,6 | 9,2 | 8,2 | 10,0 | 9,2 | 8,5 | **7,0** | **7,0** |
| Gastrointestinale Erkrankungen | 5,5 | 5,3 | 6,0 | 6,5 | 6,8 | 6,9 | 6,7 | 7,1 | 6,6 | 7,1 | 6,0 | 6,6 | 6,1 | 6,6 | 6,4 | 5,8 | **6,2** | **6,5** |
| Endokrine, Ernährungs- und  Stoffwechselerkrankungen | 0,9 | 0,9 | 1,3 | 0,9 | 1,6 | 1,8 | 2,4 | 2,8 | 3,6 | 3,6 | 5,1 | 4,4 | 6,8 | 5,5 | 5,9 | 7,0 | **3,1** | **2,6** |
| Respiratorische Erkrankungen | 2,3 | 2,2 | 1,9 | 1,9 | 2,2 | 1,9 | 2,4 | 2,4 | 3,1 | 3,6 | 2,5 | 2,9 | 2,3 | 2,7 | 1,9 | 2,4 | **2,4** | **2,5** |
| Neubildungen | 0,3 | 0,2 | 0,7 | 0,4 | 1,3 | 1,0 | 1,9 | 1,8 | 2,2 | 2,6 | 1,4 | 2,0 | 0,8 | 1,2 | 0,4 | 0,3 | **1,1** | **1,3** |
| Andere Erkrankungen ** | 40,6 | 23,7 | 38,8 | 26,1 | 32,4 | 26,6 | 29,3 | 27,7 | 28,0 | 28,4 | 24,6 | 27,4 | 20,3 | 25,2 | 18,5 | 22,0 | **30,7** | **26,3** |
| Verletzungen und Intoxikation ** | 27,4 | 46,1 | 25,0 | 36,0 | 25,6 | 30,2 | 24,3 | 23,3 | 20,0 | 16,8 | 22,1 | 18,1 | 27,2 | 23,4 | 34,7 | 33,7 | **24,7** | **28,8** |
| Muskuloskeletalle Erkrankungen | 5,5 | 6,5 | 7,9 | 8,4 | 8,3 | 7,5 | 7,7 | 6,1 | 6,2 | 4,7 | 5,6 | 3,8 | 4,1 | 3,2 | 3,6 | 2,4 | **6,2** | **5,8** |
| Cerebrovaskuläre Erkrankungen | 0,4 | 0,3 | 1,1 | 1,1 | 2,4 | 2,7 | 3,7 | 4,6 | 5,6 | 6,3 | 6,1 | 7,1 | 6,1 | 5,8 | 5,6 | 4,9 | **3,6** | **3,8** |
| Erkrankungen des Nervensystems und Sinnesorgane | 4,3 | 2,9 | 4,4 | 3,5 | 4,0 | 3,2 | 3,0 | 3,2 | 2,5 | 2,9 | 2,4 | 2,3 | 1,7 | 1,7 | 1,2 | 1,2 | **3,2** | **2,8** |
| Psychische und Verhaltens-  störungen | 3,2 | 4,5 | 3,2 | 5,4 | 3,0 | 4,6 | 2,2 | 3,0 | 1,4 | 1,3 | 1,2 | 0,9 | 1,3 | 1,1 | 1,6 | 1,4 | **2,2** | **3,1** |

F = Frauen, M = Männer

ICD = International Classification of Diseases, Gruppierung nach Ramroth

* mehrere Diagnosen pro Fall möglich

** detaillierte Aufschlüsselung siehe Tabelle 2

eTabelle 6: Dauer und Verbleib von Notaufnahmebesuchen nach Alter und Geschlecht (in %)

|  | **18-34** | | **35-44** | | **45-54** | | **55-64** | | **65-74** | | **75-84** | | **85-94** | | **95+** | | **Gesamt** | |
| --- | --- | --- | --- | --- | --- | --- | --- | --- | --- | --- | --- | --- | --- | --- | --- | --- | --- | --- |
|  | **F** | **M** | **F** | **M** | **F** | **M** | **F** | **M** | **F** | **M** | **F** | **M** | **F** | **M** | **F** | **M** | **F** | **M** |
| **Dauer bis zum ersten Arztkontakt (n = 229.219; * Informationen nur aus 8 Kliniken verfügbar, deren Anteil gültiger Werte: 90,4%)** | | | | | | | | | | | | | | | | | | |
| bis 1 Std. | 66,7 | 70,1 | 66,8 | 70,0 | 67,9 | 72,0 | 69,1 | 72,7 | 70,6 | 72,9 | 72,8 | 73,4 | 74,9 | 74,4 | 73,9 | 76,9 | **69,7** | **72,0** |
| > 1 bis 2 Std. | 22,9 | 21,3 | 23,2 | 21,5 | 22,6 | 19,5 | 21,3 | 19,0 | 20,7 | 18,7 | 18,6 | 18,7 | 17,7 | 17,8 | 19,0 | 17,7 | **21,0** | **19,7** |
| > 2 bis 3 Std. | 7,7 | 6,6 | 7,5 | 6,3 | 6,9 | 6,5 | 7,1 | 6,0 | 6,1 | 6,1 | 6,3 | 5,8 | 5,5 | 5,8 | 5,2 | 4,5 | **6,8** | **6,2** |
| > 3 bis 4 Std. | 2,7 | 2,1 | 2,6 | 2,1 | 2,6 | 2,0 | 2,4 | 2,2 | 2,5 | 2,3 | 2,3 | 2,1 | 1,9 | 1,9 | 1,9 | 0,9 | **2,5** | **2,1** |
| Mean in min | 54,0 | 50,0 | 54,1 | 50,1 | 52,6 | 48,4 | 51,7 | 47,6 | 49,6 | 47,7 | 47,8 | 46,8 | 44,8 | 45,8 | 45,3 | 41,7 | **50,8** | **48,3** |
| Median in min | 39,2 | 35,6 | 39,4 | 35,5 | 38,2 | 33,2 | 36,6 | 32,4 | 34,1 | 32,7 | 32,8 | 32,0 | 30,5 | 31,0 | 30,7 | 29,0 | **35,2** | **32,7** |
| SD in min | 46,9 | 44,6 | 46,6 | 44,5 | 46,2 | 44,5 | 46,2 | 44,6 | 45,9 | 45,0 | 45,2 | 44,2 | 43,2 | 43,7 | 43,0 | 38,6 | **46,0** | **44,5** |
| 25. Perzentil | 19,0 | 17,2 | 19,6 | 17,4 | 18,5 | 16,3 | 17,7 | 16,0 | 16,4 | 16,0 | 15,8 | 15,6 | 14,6 | 15,1 | 14,3 | 14,9 | **17,0** | **16,1** |
| 75. Perzentil | 75,0 | 69,2 | 75,0 | 69,5 | 73,0 | 66,0 | 71,3 | 64,5 | 68,7 | 64,0 | 64,5 | 63,0 | 60,3 | 61,3 | 62,7 | 57,0 | **68,8** | **64,3** |
| **Dauer in der Notaufnahme in % (n_._= 354.547; 99,5%)** | | | | | | | | | | | | | | | | | | |
| bis 1 Std. | 17,8 | 18,6 | 15,2 | 15,9 | 12,8 | 13,8 | 10,0 | 11,1 | 7,6 | 9,0 | 6,4 | 7,5 | 4,9 | 7,4 | 5,1 | 4,5 | **11,2** | **12,7** |
| > 1 bis 2 Std. | 24,9 | 28,0 | 22,8 | 25,2 | 21,9 | 22,8 | 19,7 | 20,4 | 16,4 | 18,2 | 14,9 | 16,6 | 14,7 | 15,3 | 14,6 | 21,0 | **19,6** | **21,9** |
| > 2 bis 3 Std. | 22,0 | 22,2 | 22,1 | 21,6 | 21,5 | 22,1 | 22,0 | 21,3 | 21,5 | 20,7 | 21,3 | 20,7 | 20,9 | 20,9 | 21,1 | 22,3 | **21,6** | **21,5** |
| > 3 bis 4 Std. | 14,3 | 13,1 | 15,3 | 14,6 | 16,3 | 15,1 | 16,8 | 16,4 | 18,1 | 17,3 | 18,5 | 18,0 | 19,8 | 18,6 | 20,6 | 19,7 | **16,8** | **15,8** |
| > 4 Std. | 20,9 | 18,1 | 24,6 | 22,8 | 27,5 | 26,3 | 31,5 | 30,8 | 36,4 | 34,8 | 38,9 | 37,2 | 39,7 | 37,8 | 38,6 | 32,4 | **30,8** | **28,2** |
| Mean in min | 170,8 | 160,5 | 185,1 | 177,9 | 195,9 | 192,9 | 211,8 | 209,2 | 230,2 | 223,7 | 237,7 | 230,7 | 241,6 | 231,3 | 236,5 | 217,1 | **208,0** | **198,4** |
| Median in min | 138,5 | 128,0 | 151,0 | 142,7 | 161,6 | 154,7 | 175,0 | 171,4 | 193,2 | 186,7 | 202,9 | 196,0 | 208,0 | 199,8 | 207,0 | 185,9 | **179,6** | **170,6** |
| SD in min | 147,2 | 138,6 | 153,3 | 149,1 | 157,7 | 160,6 | 163,6 | 165,9 | 170,3 | 170,3 | 168,7 | 167,8 | 166,5 | 162,6 | 165,5 | 159,5 | **162,6** | **160,0** |
| 25. Perzentil | 78,0 | 73,8 | 87,6 | 83,1 | 95,2 | 91,0 | 108,1 | 102,4 | 123,0 | 114,0 | 131,5 | 123,0 | 136,1 | 127,0 | 137,9 | 119,0 | **112,2** | **104,2** |
| 75. Perzentil | 220,0 | 204,6 | 238,5 | 229,3 | 252,0 | 246,6 | 271,3 | 268,5 | 290,4 | 284,4 | 300,0 | 292,0 | 301,4 | 293,2 | 289,9 | 272,5 | **270,4** | **261,4** |
| **Verlegungs- / Entlassungsart in % (n_._= 279.101; 78,3%)** | | | | | | | | | | | | | | | | | | |
| ambulant | 79,8 | 82,6 | 75,7 | 75,9 | 69,6 | 65,6 | 59,7 | 53,0 | 45,8 | 40,9 | 38,2 | 35,5 | 34,8 | 36,9 | 43,4 | 43,8 | **58,5** | **58,9** |
| stationär (peripher) | 18,0 | 14,4 | 21,2 | 19,7 | 25,3 | 26,9 | 32,1 | 35,8 | 43,1 | 44,7 | 49,4 | 49,6 | 53,4 | 50,9 | 49,0 | 47,0 | **34,1** | **32,1** |
| stationär - Intensiv | 1,8 | 2,4 | 2,6 | 3,5 | 4,5 | 6,6 | 7,4 | 10,1 | 10,2 | 13,3 | 11,5 | 13,7 | 10,8 | 11,2 | 6,8 | 8,6 | **6,8** | **8,1** |
| Verlegung (extern) | 0,4 | 0,6 | 0,5 | 0,9 | 0,6 | 0,9 | 0,8 | 1,1 | 0,9 | 1,1 | 0,9 | 1,2 | 1,0 | 1,1 | 0,8 | 0,6 | **0,7** | **0,9** |

F = Frauen; M = Männer

eTabelle 7:

Triage von Notaufnahmebesuchen nach Alter und Geschlecht inkl. Anteil nicht triagierter Fälle (in %)

| **n = 338.984; 95,1%** | | **Alterskategorien** | | | | | | | | |
| --- | --- | --- | --- | --- | --- | --- | --- | --- | --- | --- |
| **Geschlecht** | **Triage** | **18-34** | **35-44** | **45-54** | **55-64** | **65-74** | **75-84** | **85-94** | **95+** | **Gesamt** |
| **Frauen** | Stufe 1 - rot | 0,4 | 0,4 | 0,7 | 1,0 | 1,5 | 1,6 | 1,6 | 1,3 | 1,0 |
|  | Stufe 2 - orange | 8,2 | 10,6 | 12,5 | 15,1 | 19,1 | 20,4 | 21,3 | 23,0 | 14,9 |
|  | Stufe 3 - gelb | 32,2 | 34,0 | 34,5 | 35,9 | 39,5 | 40,2 | 39,9 | 38,1 | 36,4 |
|  | Stufe 4 - grün | 47,7 | 44,5 | 42,3 | 38,3 | 31,5 | 30,3 | 30,1 | 31,4 | 38,4 |
|  | Stufe 5 - blau | 7,4 | 6,3 | 5,4 | 4,3 | 3,4 | 2,7 | 1,9 | 1,5 | 4,7 |
|  | ohne Angabe | 4,2 | 4,2 | 4,5 | 5,3 | 4,9 | 4,9 | 5,3 | 4,7 | 4,7 |
| **Männer** | Stufe 1 - rot | 0,8 | 0,6 | 1,1 | 1,9 | 2,1 | 1,8 | 1,6 | 1,1 | 1,4 |
|  | Stufe 2 - orange | 9,2 | 12,8 | 15,8 | 18,7 | 21,4 | 20,9 | 20,5 | 20,3 | 16,1 |
|  | Stufe 3 - gelb | 25,7 | 29,3 | 32,2 | 36,0 | 40,0 | 41,4 | 41,3 | 39,3 | 33,9 |
|  | Stufe 4 - grün | 53,9 | 47,0 | 41,0 | 34,1 | 28,0 | 28,0 | 29,0 | 33,5 | 39,2 |
|  | Stufe 5 - blau | 6,2 | 5,7 | 4,7 | 3,7 | 3,0 | 2,4 | 2,2 | 1,4 | 4,3 |
|  | ohne Angabe | 4,3 | 4,5 | 5,2 | 5,7 | 5,3 | 5,5 | 5,4 | 4,4 | 5,1 |

**eBox Stärken und Schwächen**

Wesentliche Stärke dieser Arbeit ist unsere hohe Fallzahl mit fast 360.000 Patienten aus insgesamt elf deutschlandweit verteilten Notaufnahmen mit unterschiedlichen Versorgungsstufen. Hinzu betrachteten wir altersspezifische Daten, wofür ebenfalls diese große Fallzahl hilfreich war. Saisonale Schwankungen beeinflussen die Ergebnisse nicht, da wir ein komplettes Kalenderjahr untersuchten. Insgesamt handelt es sich um eine der ersten solchen Analysen aus Deutschland, da nur wenige Daten dazu in der Literatur zu finden sind. Zudem ermöglichte das AKTIN-Notaufnahmeregister eine Auswertung standardisiert erfasster Daten.

Trotzdem ist die Hauptlimitation eine teilweise hohe Anzahl von Missings bei einzelnen Variablen (z.B. 35,7% bei der Dauer bis zum ersten Arztkontakt, weil dafür drei Notaufnahmen aufgrund fehlender Daten ausgeschlossen werden mussten; von diesen 8 Kliniken lagen allerdings 90,4% an Daten vor). Weiterhin lagen auch zu etwa 56.000 Besuchen keine Diagnosen vor. Aufgrund einer hohen Zahl fehlender Werte bei wichtigen Variablen wurden vorab bereits drei von 14 Kliniken ausgeschlossen. Insgesamt müssen fehlende Werte jedoch nicht zwingend zu einer systematischen Verzerrung führen, wie wir durch den Vergleich triagierte vs. nicht triagierte Notaufnahmepatienten exemplarisch näher untersucht haben. So wurden 4,7% der Männer und 5,1% der Frauen nicht triagiert und in keiner Altersgruppe überstieg dieser Anteil 5,7% (s. eTabelle 7). Auch der Anteil ambulanter Fälle war identisch (nicht triagiert vs. triagiert jeweils 59%), auch wenn ein größerer Anteil nicht triagierter Fälle direkt auf eine Intensivstation aufgenommen wurde (16,3% vs. 6,9%) bzw. notarztbegleitet kam (13,2% vs. 8,5%). Es zeigt sich somit eine leichte Tendenz, dass kränkere Patienten häufiger nicht ersteingeschätzt werden. Bei anderen Variablen dürften mögliche systematische Verzerrungen durch Missings allerdings noch weniger relevant sein. Eigene Auswertungen einer der eingeschlossenen Notaufnahmen über einen längeren Zeitraum zeigen z.B. bei der Einweisungsart im Zeitverlauf zunehmend vollständigere Daten, ohne dass es relevante Hinweise auf eine systematische Verzerrung unvollständig erfasster Zeiträume gab. Insgesamt waren jedoch notaufnahmespezifische Auswertungen in dem abgestimmten Analyseplan nicht vorgesehen bzw. waren auch nicht Ziel dieser Arbeit. Zusätzlich lagen uns keine Angaben zu weiteren klinischen Parametern wie Labor- oder Radiologiebefunden vor, wodurch z.B. die Angemessenheit einer Versorgung in der Notaufnahme nicht ermittelt werden kann. Auch Daten zu Einschränkungen der Mobilität oder Kognition sowie Pflegegrade bzw. ob die Patienten aus dem Pflegeheim kamen, sind im AKTIN-Notaufnahmeregister nicht erfasst. Zudem waren Informationen zur weiteren Versorgung bei stationären Fällen nicht verfügbar.
